# Gender-neutral vs. gender-specific strategies in school-based HPV vaccination programs: a systematic review and meta-analysis

**DOI:** 10.3389/fpubh.2025.1460511

**Published:** 2025-02-18

**Authors:** Nutthaporn Chandeying, Puttichart Khantee, Sirada Puetpaiboon, Therdpong Thongseiratch

**Affiliations:** ^1^Department of Obstetrics and Gynecology, Faculty of Medicine Vajira Hospital, Navamindradhiraj University, Bangkok, Thailand; ^2^Department of Pediatrics, Faculty of Medicine, Prince of Songkla University, Songkhla, Thailand

**Keywords:** HPV vaccination, gender-neutral strategies, gender-specific strategies, school-based interventions, vaccination uptake

## Abstract

**Introduction:**

This systematic review and meta-analysis evaluated whether gender-neutral (GN) or gender-specific (GS) strategies more effectively enhanced knowledge, intention, and uptake of HPV vaccination among students in educational settings.

**Methods:**

A comprehensive literature search of PubMed, Scopus, Web of Science, and Cochrane Library identified 17 randomized controlled trials encompassing 22,435 participants (14,665 females, 7,770 males). Random-effects models were used to calculate standardized mean differences (SMDs) for knowledge and intention, and risk differences for vaccination uptake.

**Results:**

GN strategies achieved higher improvements in knowledge (SMD = 0.95) and intention (SMD = 0.59) compared with GS (SMD = 0.68 for knowledge, SMD = 0.14 for intention), and displayed a greater increase in uptake (5.7% versus 2.5% in GS), although this uptake difference was not statistically significant. Heterogeneity was more pronounced for knowledge outcomes and moderate for GS uptake results.

**Discussion:**

Despite GN approaches seemingly offering more robust enhancements in HPV-related knowledge and vaccination intention, additional research with robust designs and longer follow-up is required to determine whether GN interventions definitively outperform GS strategies in achieving statistically significant increases in actual vaccination uptake.

## 1 Introduction

Human papillomavirus (HPV) is a significant global health concern, responsible for a substantial burden of disease worldwide (1–3). HPV is the primary cause of several cancers, including cervical, oropharyngeal, anal, and genital cancers (4). The introduction of HPV vaccines has shown substantial promise in reducing the incidence of these malignancies, particularly cervical cancer (5, 6). The World Health Organization (WHO) has set an ambitious goal to eliminate cervical cancer as a public health problem by achieving 90% HPV vaccination coverage among girls by the age of 15, coupled with high screening and treatment rates (7, 8).

In low- and middle-income countries (LMICs), the introduction and scale-up of HPV vaccination have been particularly challenging due to limited healthcare resources, cultural stigma, and logistical constraints in delivering multi-dose vaccines. From a meta-analysis of HPV vaccine coverage during the period 2006–2020, the pooled estimate of vaccination uptake in 24 LMICs was 61.69%, although this varied considerably across countries (9). Despite WHO’s efforts, many regions have not achieved the desired vaccination coverage, primarily due to barriers such as vaccine hesitancy, lack of awareness, and limited access to healthcare services (8–10). Despite these variations, coverage rates in many LMICs still lag behind those in high-income countries. Addressing these obstacles is essential to fully realize the potential of HPV vaccines and to make significant strides toward the elimination of cervical cancer (10, 11).

Various interventions have been developed to improve HPV vaccination rates, with school-based programs emerging as particularly effective (12). Schools, colleges, and universities provide unique opportunities to reach adolescents and young adults in a structured environment conducive to health education and vaccination campaigns. School-based interventions have the advantage of integrating vaccination programs into existing health curricula, ensuring wider reach and accessibility. These interventions can leverage the trust and influence that educational institutions have over students, facilitating higher vaccination uptake (13–16).

Gender-specific (GS) strategies primarily target females, emphasizing the prevention of cervical cancer through focused educational sessions and health promotion activities (17). These interventions have demonstrated success in raising awareness and increasing vaccination rates among females, contributing significantly to the prevention of cervical cancer (18, 19). However, this approach has a notable limitation: it does not address the significant risk of HPV-related cancers in males, such as oropharyngeal and anal cancers. By focusing solely on females, GS strategies miss the opportunity to educate and protect the entire population at risk, thereby potentially underutilizing the full potential of HPV vaccination programs (20, 21).

In our review, we define GS strategies as those primarily or exclusively targeting females for HPV-related education, motivation, or vaccination campaigns. Although some GS programs may employ principles that could be considered ‘gender-responsive’ or ‘gender-transformative,’ our focus was on the overarching approach of directing HPV vaccination interventions specifically at female students rather than undertaking broader structural or systemic gender transformations. Conversely, GN strategies were those aiming to inform and involve all genders, often emphasizing male and female vaccination equally (22, 23).

The underlying hypothesis of GN strategies is that by targeting a wider demographic, these interventions can foster a more inclusive and widespread understanding of HPV prevention. This inclusivity is expected to lead to higher vaccination rates across all genders, thus maximizing public health benefits. Additionally, we hypothesize that the effect of GN strategies on females, even though not specifically focused on them, may be better than that of GS strategies (24, 25). This is because GN strategies place less emphasis on sexual activity and leverage the behavioral economic nudge of the “default” that all children should be vaccinated, which can reduce stigma and encourage vaccination uptake (26, 27). Emerging evidence supports the effectiveness of GN strategies in promoting vaccine equity and inclusivity, suggesting they may be more effective in reducing the overall burden of HPV-related cancers (28). By engaging all genders, GN strategies hold the potential to create a more holistic and effective public health response to HPV (29).

While one might initially assume that GN or GS strategies focus solely on providing vaccines to all genders or only females (30), it is intriguing to shift the focus toward strategies that go beyond merely offering vaccination. Instead, these strategies aim to improve vaccination uptake through educational and promotional efforts. This shift highlights the importance of interventions designed to enhance understanding and acceptance of HPV vaccination, thereby increasing actual vaccination rates. The objectives of this systematic review and meta-analysis are twofold: first, to assess the effectiveness of GN versus GS strategies in enhancing knowledge and attitudes toward HPV-related cancer prevention in educational settings; and second, to evaluate whether GN or GS strategies result in higher HPV vaccination rates among students in schools, colleges, and universities. To provide a comprehensive understanding, we will separate the analysis of outcomes into three categories: outcomes for all genders comparing GN versus GS strategies, outcomes for females comparing GN versus GS strategies, and outcomes for males comparing GN versus GS strategies. Through a comprehensive analysis of randomized controlled trials (RCTs) conducted in these settings, this study seeks to provide robust evidence to inform future HPV vaccination policies and programs, with the ultimate goal of optimizing vaccination uptake and reducing HPV-related cancer incidence globally.

## 2 Materials and methods

### 2.1 Study design

This systematic review and meta-analysis were conducted to rigorously evaluate the effectiveness of GS and GN strategies implemented in educational settings for improving knowledge, attitudes, and vaccination uptake related to HPV prevention. The research protocol was proactively registered with PROSPERO, the International Prospective Register of Systematic Reviews (ID: CRD42024566215), and the Open Science Framework (OSF), accessible at https://osf.io/qjbmu/ (accessed on 2 Jan 2025), to underscore our commitment to methodological rigor and transparency. Our methods and the reporting of results were strictly in line with the detailed recommendations provided in the Preferred Reporting Items for Systematic Reviews and Meta-Analyses (PRISMA) 2020 guidelines (31). We also adhered to the methodological standards set forth in the Cochrane Handbook for Systematic Reviews of Interventions (32).

### 2.2 Eligibility criteria

Eligibility for inclusion in this study was limited to peer-reviewed articles written in English that conformed to the PICOS (Population, Intervention, Comparator, Outcome, Study Design) framework as follows (33, 34).

#### 2.2.1 Population (P)

We included studies involving adolescents and young adults in educational settings, such as schools, colleges, and universities. This ensured the target demographic was relevant to the interventions aimed at increasing HPV vaccination uptake in these specific environments. Studies focusing on non-educational settings or involving populations outside of the specified age groups were excluded.

#### 2.2.2 Intervention (I)

This review focused on both GS HPV prevention strategies targeted exclusively at females and GN HPV prevention strategies that were not targeted at a specific gender. The interventions encompassed a variety of strategies, including educational programs, web-based education, and motivational interviewing, all designed to enhance knowledge, attitudes, and vaccination rates. We included both onsite interventions and those delivered via web platforms or other digital means. Excluded were interventions that did not involve an active educational component, such as the passive distribution of materials like brochures or posters.

#### 2.2.3 Comparator (C)

Included studies had to compare the effectiveness of school-based strategies against standard practices or control conditions that did not employ the targeted HPV prevention strategies. This could include usual care, waiting list controls, or different types of interventions. Studies using comparators that involved non-educational or non-behavioral strategies, such as pharmacological interventions or structural changes within healthcare settings, were excluded.

#### 2.2.4 Outcomes (O)

The primary outcomes of interest were the effectiveness of GS and GN strategies in improving knowledge, attitudes toward HPV-related cancer prevention, and HPV vaccination uptake. This included specific measures of knowledge improvement, changes in attitudes, and actual vaccination rates. Studies that did not directly report on these outcomes, or focused on indirect measures such as general health outcomes or non-specific educational metrics, were excluded from this review. This focus ensured that our analysis directly assessed the impact of the interventions on tangible vaccination-related outcomes.

#### 2.2.5 Study design (S)

We included only RCTs in this review, as they provide the highest level of evidence for assessing the efficacy of interventions. This choice was made to maintain the rigor and specificity of the evidence evaluated in this meta-analysis. Excluded were non-randomized studies, observational studies, case reports, review articles, and qualitative studies.

### 2.3 Search strategy

We conducted a comprehensive literature search using PubMed, Scopus, Web of Science, and the Cochrane Library on 3 June 2024. The search strategy was designed to include terms related to ‘HPV vaccination’, ‘communication’, and ‘educational settings’. Keywords and MeSH terms were used in various combinations: (HPV OR ‘human papillomavirus’) AND (vaccin* OR immuni* OR ‘vaccine uptake’) AND (gender OR sex) AND (education OR ‘school-based’ OR ‘college-based’ OR ‘university-based’). The complete search strategies are provided in the Supplementary material. Filters were initially applied to restrict the search to studies published in English from January 2000 to December 2023. However, since the first HPV vaccine became available in 2006, we focused on studies published from January 2006 to December 2023. Additional sources included reference lists of relevant articles and consultations with organizations. The last search was conducted on 3 June 2024.

### 2.4 Study selection

The reference lists of relevant systematic reviews and primary studies were reviewed to identify additional studies. NC and TT independently conducted an initial screening of titles and abstracts using Rayyan,^1^ a systematic review software, to identify studies potentially meeting the eligibility criteria. Full-text articles of these potentially eligible studies were then thoroughly evaluated for final inclusion by a research assistant along with NC and TT. Discrepancies were resolved through discussion or by consulting a third reviewer to ensure the accuracy and reliability of the selection process. No automation tools were used beyond the initial screening in Rayyan (35).

### 2.5 Data extraction

Data extraction was performed independently by two reviewers using a standardized form to ensure a comprehensive and consistent approach. Extracted data included general study characteristics such as study design, duration, specific details about the interventions (e.g., type of intervention and delivery method), characteristics of the study sample (including demographic information and setting), and relevant outcome data necessary for calculating effect sizes.

For studies that reported both intention-to-treat and per-protocol analyses, intention-to-treat data were prioritized to maintain consistency and robustness in our analysis (36). In studies employing cluster sampling, the sample sizes were adjusted based on the reported design effect and intracluster correlation coefficients to accurately reflect the impact of this study (37). All extracted data were systematically organized and recorded in Microsoft Excel. Data extraction and coding were performed by a research assistant, overseen by NC and TT, to ensure the accuracy and reliability of the data handling process. When necessary, authors of the studies were contacted to clarify or obtain additional data that were not available from the publications. Discrepancies in data extraction were resolved through discussion or consultation with a third reviewer, ensuring the integrity of the data collected. No automation tools were used.

### 2.6 Quality assessment

To ascertain the credibility of the cluster randomized trials included in our systematic review, we employed the revised Cochrane risk-of-bias tool for randomized trials (RoB 2), specifically tailored for cluster-randomized trials (38). This comprehensive tool enabled us to assess bias across several domains: the randomization process, deviations from intended interventions, missing outcome data, measurement of the outcome, and selection of the reported result. Each domain was meticulously examined to determine the level of bias present, with judgments categorized as ‘low risk’, ‘some concerns’, or ‘high risk’. The evaluation of each domain was conducted independently by NC and TT to enhance objectivity, with any discrepancies resolved through discussion or consultation with a third reviewer. Additionally, to explore potential publication bias, we utilized funnel plots, which provided a visual assessment of the symmetry in the distribution of effect sizes, further validating the robustness of our meta-analytical findings (39).

### 2.7 Data synthesis and analysis

Data were synthesized quantitatively using meta-analysis methods where appropriate, employing the Comprehensive Meta-Analysis software version 4 (Biostat, Englewood, NJ, USA) to facilitate statistical analysis. Effect sizes were calculated using random-effects models to account for variability between studies. We used the risk difference of vaccination uptake as the main effect measure, represented as the mean percentage increase in vaccination uptake. For knowledge and intention outcomes, standardized mean differences (SMDs) were used to summarize the effect sizes. To accommodate potential variability across the included studies, we utilized a random-effects model, which is better suited for handling the expected heterogeneity. Subgroup analyses were conducted to compare GN versus GS strategies for knowledge, intention, and HPV vaccination uptake.

The extent of this heterogeneity was quantitatively assessed using the I^2^ statistic, with cut-off values interpreted as follows: 0–40% may indicate low heterogeneity, 30–60% may indicate moderate heterogeneity, 50–90% may indicate substantial heterogeneity, and 75–100% may indicate considerable heterogeneity, as recommended by the Cochrane Handbook for Systematic Reviews of Interventions. Statistical significance was indicated by a *p*-value of less than 0.05. Uncertainty was expressed using 95% confidence intervals. Results are graphically presented using forest plots to visually represent the effect sizes and their confidence intervals (32).

## 3 Results

**Figure 1:**
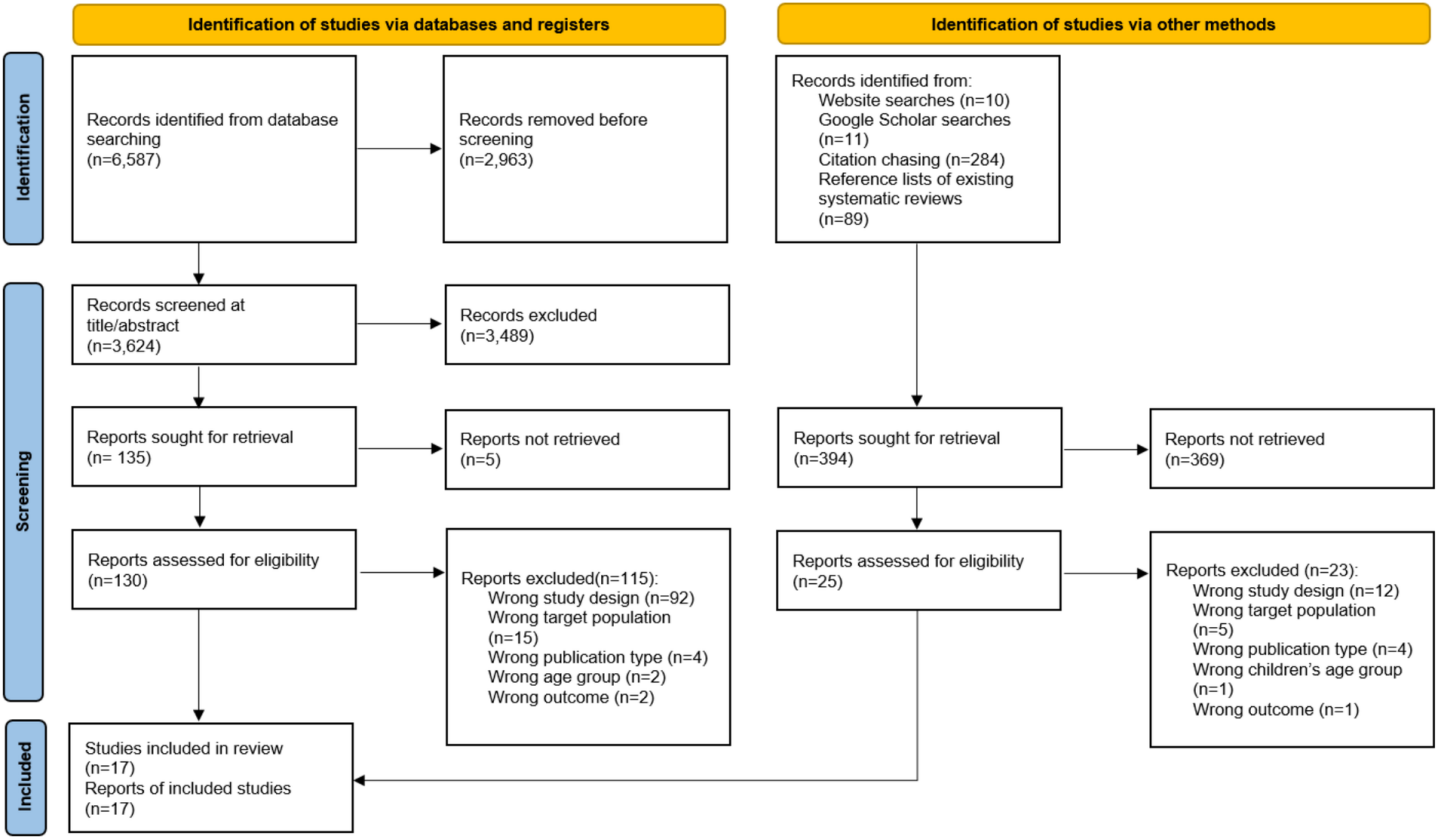
PRISMA 2020 flow diagram.

Figure 1 presents the flow diagram of the study selection process. Initially, searches across various electronic databases yielded 6,587 studies. After removing duplicates, 3,624 studies remained for further examination. Screening of titles and abstracts led to the exclusion of 3,489 studies, resulting in 135 full-text articles retrieved for detailed evaluation. Following a thorough review, studies that did not meet the inclusion criteria were excluded. Additionally, a study identified through alternative methods, such as website searches, Google Scholar searches, citation chasing, and references lists of existing systematic reviews, was added. Ultimately, a total of 17 studies were included in the final analysis.

All 17 studies included in this meta-analysis are randomized controlled trials (RCTs). The total sample size across these studies is 22,435 participants. Of these, 14,665 are females and 7,770 are males. The studies employed either gender-neutral (GN) or gender-specific (GS) strategies. Specifically, 10 studies used GN strategies, involving 13,678 participants (40–46), and 7 studies used GS strategies, encompassing 8,757 participants (31–40, 47–50).

### 3.1 Intervention strategies across studies

The studies were conducted in various educational settings, including schools, colleges, and universities (Table 1). Several studies targeted high school and secondary school students (35, 50, 51). Most of the studies focused on college and university students, utilizing the structured environment to deliver educational interventions (40, 44–46, 48–50, 52, 53). The age of participants varied across the studies, typically reflecting the educational setting. For high school students, participants were generally adolescents aged 12–17 years (35, 50, 51). For college and university students, participants were typically young adults aged 18–24 years (40, 44–46, 48–50, 52, 53).

**Table 1:** Summary of included studies.

Study	Location	Population	Sample size	Intervention	Comparison	Duration/Follow-up	Outcomes
Baxter et al. (40)	Canada	Female university students (GS)	Total = 193, *I* = 98, *C* = 95	Tailored HPV message for sexually inexperienced women	Detailed HPV message, Control	Immediate	Intention
Bennett et al. (48)	USA	Female university students (GS)	Total = 661, *I* = 330, *C* = 331	MeFirst tailored online educational intervention	Standard CDC factsheet	3 months	Knowledge, Uptake
Davies et al. (49)	Australia	Secondary school students (GN)	Total = 6,965, *I* = 3,485, *C* = 3,480	Complex intervention (education and distraction, decisional support, logistical strategies)	Usual practice	End of school year	Knowledge
Doherty et al. (41)	USA	College students (GN)	Total = 119, *I* = 60, *C* = 59	Web-based HPV educational intervention	Control	1 month	Knowledge, Intention
Grandahl et al. (52)	Sweden	Upper secondary school students (GN)	Total = 751, *I* = 376, *C* = 375	Face-to-face structured information about HPV by school nurses	Regular health interview	3 months	Intention
Hopfer et al. (42)	USA	Female college students (GS)	Total = 404, *I* = 202, *C* = 202	Narrative intervention (peer-only, medical expert-only, combined peer-expert)	Informational video, campus website, no message	2 months	Uptake
Kim et al. (50)	USA	Korean American college women (GS)	Total = 104, *I* = 52, *C* = 52	Storytelling video intervention using mobile, web-based technology	Information-based written material	2 months	Uptake
McKeever et al. (43)	USA	College-age women (GS)	Total = 73, *I* = 42, *C* = 31	Educational program about cervical cancer, HPV infection, and HPV vaccine	Educational program offered after 1 month	1 month	Knowledge, Intention
Merzouk et al. (53)	USA	High school students (GN)	Total = 626, *I* = 313, *C* = 313	HPV educational DVD plus health class	Health class only	Immediate	Knowledge
Nadarzynski et al. (44)	UK	Female university students (GS)	Total = 606, *I* = 303, *C* = 303	Information about cervical cancer and HPV (control, control + HPV, control + risk factors, control + both)	Control	1 week	Knowledge
Perez et al. (45)	USA	College-aged women (GS)	Total = 62, *I* = 31, *C* = 31	Information–motivation–behavioral skills (IMB) intervention	Attention control	1 month	Knowledge, Intention
Si et al. (46)	China	Female university students (GS)	Total = 3,739, *I* = 1936, *C* = 1803	10-min online IMB model-based education daily for 7 days	Health tips unrelated to HPV	Immediate	Knowledge, Intention
Steckelberg et al. (56)	Germany	Vocational school girls (GS)	Total = 105, *I* = 53, *C* = 52	Standard leaflet supplemented with numerical information on cancer risk and HPV vaccination benefits	Standard leaflet without numerical data	Immediate	Knowledge
Stock et al. (51)	USA	College students (GN)	Total = 238, *I* = 125, *C* = 113	Information on HPV, oral sex, and oral cancer	No information	Immediate	Knowledge, Intention
Tull et al. (62)	Australia	Parents of year 7 students (GN)	Total = 4,386, *I* = 2,834, *C* = 1,552	SMS reminder to parents (motivational vs. self-regulatory)	No SMS	End of school year	Uptake
Wang et al. (55)	China	Female first-year college students (GS)	Total = 449, *I* = 235, *C* = 214	7 days of HPV-related web-based education	Popular science education (not HPV-related)	3 months	Knowledge, Intention
Zhang et al. (54)	China	Female freshmen (GS)	Total = 946, *I* = 532, *C* = 414	7-day web-based health education on HPV and HPV vaccines	Non-HPV related materials	1 month	Knowledge, Intention

GN, Gender neutral; GS, Gender specific; I, Intervention; C, Control.

The interventions varied in their approach and delivery methods. Some studies implemented tailored educational interventions that addressed specific knowledge gaps about HPV and its vaccines (40, 44–46, 49, 50, 52, 54). Others used narrative and storytelling methods to make the information more relatable and engaging for the participants (44–46, 48). Interventions also included components such as motivational interviewing, decisional support, and logistical strategies to facilitate vaccination (40, 50, 51, 55). The duration of interventions ranged from single sessions to daily sessions over a week, with follow-up periods varying from immediate post-intervention to several months. The use of technology was a common feature in the interventions, enhancing the delivery and engagement of educational content. Many studies used web-based platforms to deliver educational content and interventions (43, 44, 46, 48, 49, 52, 54). Some interventions utilized mobile applications for delivering content, reminders, and tracking vaccination status (40, 44, 46). Additionally, SMS reminders were used to prompt parents and students about vaccination appointments and educational content (50, 51, 55).

The studies employing GN strategies targeted both males and females, aiming to provide a comprehensive understanding of HPV and its associated risks across genders. These studies often used inclusive educational materials and interventions that addressed the full spectrum of HPV-related health risks, thus ensuring a broader reach and impact. For instance, GN strategies included web-based education and mobile applications that provided interactive and engaging content for all students (40–46). Additionally, GN interventions frequently involved peer education and storytelling methods to make the information relatable and engaging for both genders (44–46, 48). In contrast, GS strategies focused primarily on female participants, emphasizing the prevention of cervical cancer through targeted educational sessions and health promotion activities. These studies often highlighted the importance of HPV vaccination for preventing cervical cancer, with interventions designed to address specific knowledge gaps and misconceptions among women (40, 44, 46, 48–50, 52, 54). GS strategies also utilized tailored educational interventions and motivational interviewing techniques to increase vaccination intentions and uptake among female students (40, 44, 46, 49, 50, 52, 54).

The comparison groups in these studies typically received either standard or minimal educational interventions about HPV and its vaccination. In some studies, the control groups received standard health education materials, such as CDC factsheets or regular health class content (40, 44, 46, 48–52, 54). Other control groups received no additional information beyond what was typically provided in their educational settings (43, 44, 46, 48, 55). The aim of these comparisons was to evaluate the added benefit of the tailored, technologically enhanced, and nudge-based interventions over the standard or minimal educational approaches.

The outcomes assessed in the studies varied but focused on three primary areas: knowledge, intention to vaccinate, and actual vaccination uptake. Most studies evaluated the participants’ knowledge about HPV, its related diseases, and the benefits of vaccination. The interventions generally led to significant improvements in HPV-related knowledge compared to controls (40–44, 46, 48, 50, 53–56). Several studies measured the intention to get vaccinated as an intermediate outcome. Interventions that included motivational and educational components were effective in increasing participants’ intention to receive the HPV vaccine (40, 44, 46, 49, 50, 52, 54). Actual vaccination uptake was assessed in studies that had longer follow-up periods. Both GN and GS strategies showed effectiveness in increasing vaccination rates, but GN strategies demonstrated a broader impact by also addressing male vaccination, thereby contributing to higher overall uptake rates (44, 46, 48–50, 52–55).

### 3.2 Meta-analysis

#### 3.2.1 HPV-related knowledge

The impact of interventions on HPV-related knowledge was assessed across multiple studies, with a total of 13 studies included in the analysis. The results from the fixed-effect analysis for both GN and gender-specific GS strategies are as follows:

For GN strategies, the pooled effect size from 5 studies was 0.954 (95% CI, 0.537–1.371) with a standard error of 0.213 and a variance of 0.045. The Z-value for the test of null was 4.482, with a *p*-value of <0.001, indicating a statistically significant improvement in knowledge. The heterogeneity among the studies was significant, with a Q-value of 88.16 (df = 4, *p* < 0.001) and an I^2^ value of 95.46%, indicating substantial heterogeneity.

For GS strategies, the pooled effect size from 8 studies was 0.226 (95% CI, −0.185–0.638) with a standard error of 0.210 and a variance of 0.044. The *Z*-value for the test of null was 1.078, with a *p*-value of 0.281, indicating no statistically significant improvement in knowledge. The heterogeneity among these studies was also significant, with a *Q*-value of 202.07 (df = 7, *p* < 0.001) and an I^2^ value of 96.54%, indicating considerable heterogeneity.

**Figure 2:**
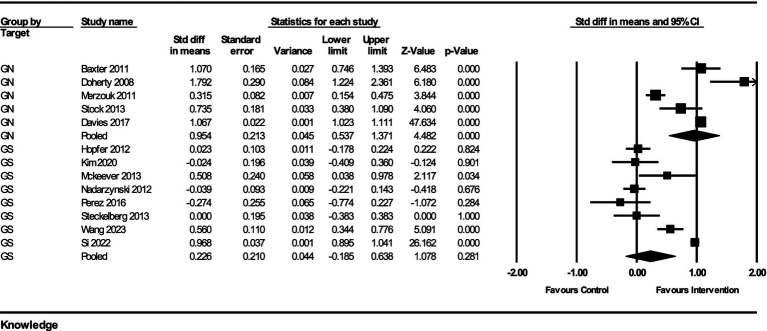
Forest plot of the effects on HPV-related knowledge of GN vs. GS.

The subgroup analysis comparing the GN and GS strategies revealed a significant difference between the two groups. The *Q*-value for the subgroup difference was 5.914 (df = 1, *p* = 0.015). This indicates that GN strategies had a significantly greater impact on improving HPV-related knowledge compared to GS strategies (Figure 2).

#### 3.2.2 HPV vaccination intention

The impact of interventions on the intention to receive the HPV vaccine was assessed in several studies. The results from the fixed-effect analysis for both gender-neutral (GN) and gender-specific (GS) strategies are summarized below:

For GN strategies, the pooled effect size from 1 study was 0.593 (95% CI, 0.242–0.944) with a standard error of 0.179 and a variance of 0.032. The *Z*-value for the test of null was 3.313, with a *p*-value of 0.0009, indicating a statistically significant improvement in vaccination intention. There was no heterogeneity among the GN studies, as the *Q*-value was 0 (df = 0, *p* = 1) and the *I*^2^ value was 0%.

For GS strategies, the pooled effect size from 5 studies was 0.141 (95% CI, 0.006–0.282) with a standard error of 0.072 and a variance of 0.005. The *Z*-value for the test of null was 1.969, with a *p*-value of 0.049, indicating a marginally significant improvement in vaccination intention. The heterogeneity among these studies was minimal, with a *Q*-value of 0.923 (df = 4, *p* = 0.921) and an *I*^2^ value of 0%.

**Figure 3:**
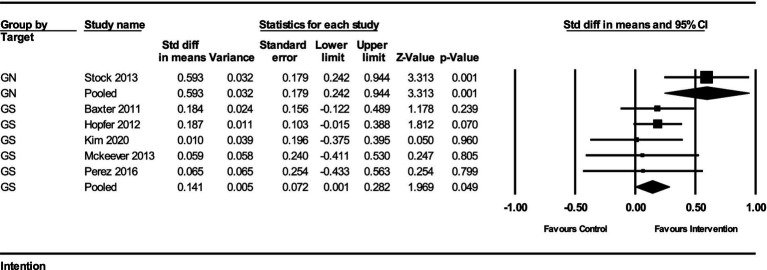
Forest plot of the effects on HPV vaccination intention of GN vs. GS.

The subgroup analysis comparing the GN and GS strategies revealed a significant difference between the two groups. The *Q*-value for the subgroup difference was 5.494 (df = 1, *p* = 0.019). This indicates that GN strategies had a significantly greater impact on improving HPV vaccination intention compared to GS strategies (Figure 3).

#### 3.2.3 HPV vaccination uptake

The impact of interventions on HPV vaccination uptake was assessed using risk difference as the effect measure. The results from the fixed-effect analysis for both gender-neutral (GN) and gender-specific (GS) strategies are summarized below:

For GN strategies, the pooled risk difference from 2 studies was 0.057 (95% CI, 0.028–0.087), indicating a 5.7% increase in vaccination uptake (standard error = 0.015, variance = 0.00022). The *Z*-value for the test of null was 3.841, with a *p*-value of 0.00012, indicating a statistically significant improvement in vaccination uptake. There was no significant heterogeneity among the GN studies (*Q*-value = 0.559, df = 1, *p* = 0.455, *I*^2^ = 0%).

For GS strategies, the pooled risk difference from 5 studies was 0.025 (95% CI, −0.009–0.059), indicating a 2.5% increase in vaccination uptake (standard error = 0.017, variance = 0.00030). The *Z*-value for the test of null was 1.444, with a *p*-value of 0.149, suggesting a non-significant improvement in vaccination uptake. The heterogeneity among these studies was substantial (*Q*-value = 19.855, df = 4, *p* = 0.00053, *I*^2^ = 79.85%).

**Figure 4:**
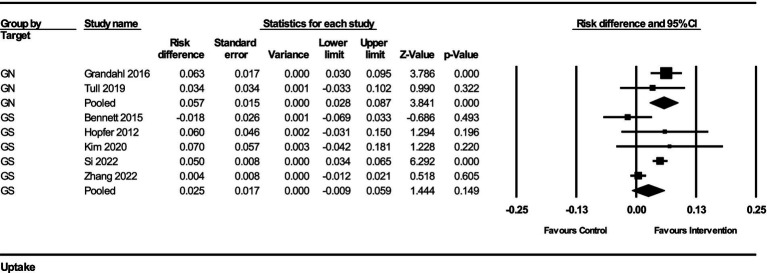
Forest plot of the effects on HPV vaccination uptake of GN vs. GS.

The subgroup analysis comparing the GN and GS strategies revealed no significant difference between the two groups (*Q*-value = 2.046, df = 1, *p* = 0.153). This indicates that while GN strategies showed a more substantial and statistically significant improvement in HPV vaccination uptake, the difference between GN and GS strategies was not statistically significant in this analysis (Figure 4).

### 3.3 Publication bias

**Figure 5:**
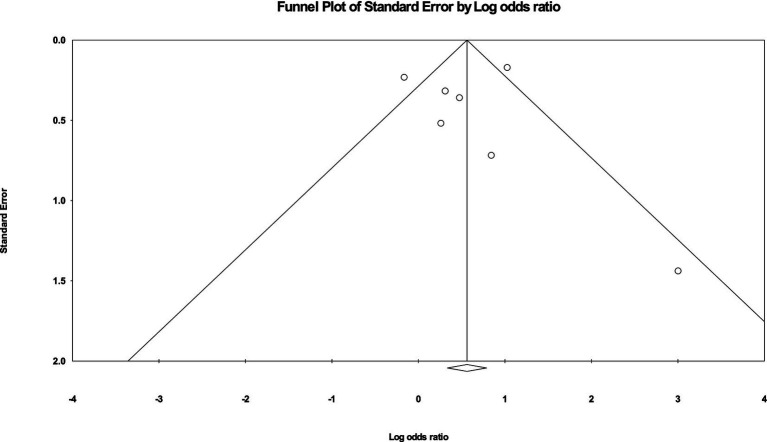
Funnel plot with symmetrical spread of effect sizes around the mean effect size.

Visual inspection of the funnel plot (Figure 5) did not reveal significant signs of publication bias, which supports the credibility of the meta-analysis findings. The effect sizes were distributed relatively symmetrically across the studies, suggesting that there was no systematic bias skewing the results. Most effect sizes fell within the funnel, indicating a uniform distribution. A few effect sizes that fell outside the funnel did so symmetrically on both sides of the mean, further reducing concerns about potential bias. This symmetry implies that both smaller and larger studies contributed evenly to the overall analysis, indicating that the meta-analytical conclusions are robust and reliable across different study sizes and conditions.

### 3.4 Risk of bias analysis

**Figure 6:**
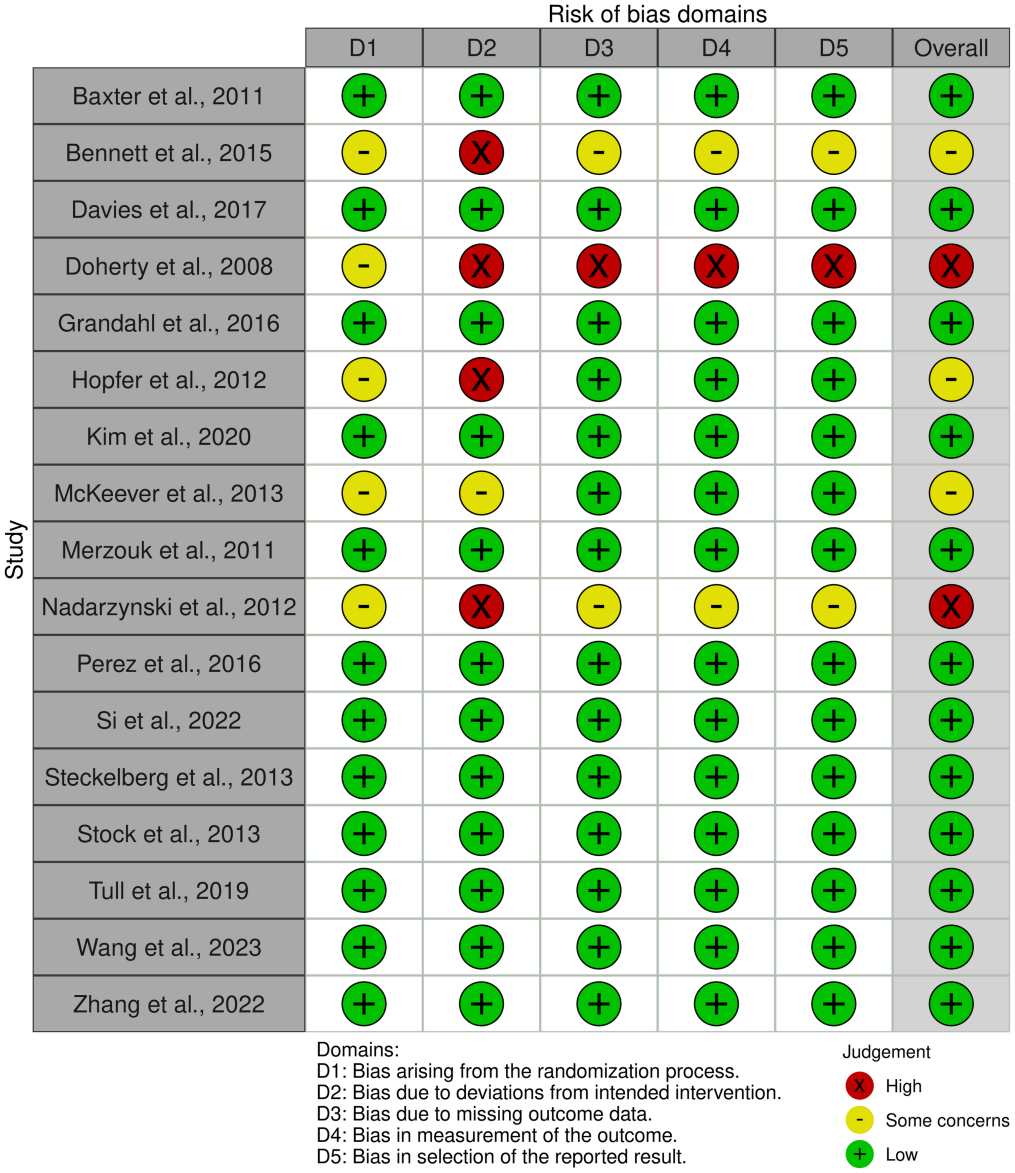
Risk of bias plots.

The risk of bias was assessed across all 17 studies using the Cochrane Collaboration’s tool for randomized controlled trials (RoB 2). The assessment covered five domains: bias arising from the randomization process, bias due to deviations from intended interventions, bias due to missing outcome data, bias in measurement of the outcome, and bias in selection of the reported result. Overall, the risk of bias assessment revealed that most studies had low risk in several domains, with some concerns primarily arising from randomization and deviations from intended interventions. This assessment underscores the robustness and reliability of the meta-analytic findings, although the identified risks highlight areas for potential improvement in future research designs (Figure 6).

## 4 Discussion

This systematic review and meta-analysis evaluated the effectiveness of GN versus GS strategies in enhancing knowledge, intention, and uptake of HPV vaccination among students in educational settings. Our analysis, which included 17 RCTs with a total sample size of 22,435 participants, revealed that both GN and GS strategies effectively improve HPV-related knowledge and vaccination intention. However, GN strategies demonstrated a more significant impact on vaccination uptake, suggesting a broader reach in public health interventions.

The analysis revealed that GN strategies significantly improve HPV-related knowledge compared to GS strategies. The SMD for GN strategies was 0.95, indicating a substantial increase in knowledge levels. This finding is consistent with previous research suggesting that inclusive educational interventions can enhance understanding across diverse populations (57, 58). However, the high heterogeneity observed in knowledge outcomes (*I*^2^ = 95.46% for GN and 96.54% for GS) suggests variability in intervention delivery and educational settings, which may influence the effectiveness of knowledge dissemination. Both GN and GS strategies were effective in increasing vaccination intention, with GN strategies showing a more pronounced effect (SMD = 0.59) compared to GS strategies (SMD = 0.14). This aligns with earlier studies indicating the critical role of motivational and educational components in shaping vaccination intentions (58). The minimal heterogeneity observed in the GS group (*I*^2^ = 0) suggests a consistent effect of these interventions on vaccination intentions, while the GN group exhibited no heterogeneity, reflecting a uniform impact across the included studies.

It is important to note that in this review, we defined GS strategies as those primarily or exclusively targeting female populations for HPV vaccination and education. Although some GS interventions may contain elements of gender responsiveness—by acknowledging and accommodating distinct needs of women—this does not necessarily mean they are fully gender-transformative, which would involve actively challenging gender norms and power imbalances. Similarly, GN strategies, while often involving both male and female participants, may still require further refinements to align with gender-transformative frameworks in certain cultural or educational contexts.

The findings from our study suggest that school-based HPV vaccination programs can improve knowledge about HPV infection and HPV vaccination among female students. This aligns with previous systematic reviews and meta-analyses, which have highlighted the effectiveness of educational interventions in increasing knowledge and altering perceptions about HPV and cervical cancer (57–59). Ampofo et al. (58) conducted a meta-analysis focusing on the effectiveness of school-based education for improving knowledge and perceptions of cervical cancer and HPV among female students. Their study found that while knowledge about cervical cancer and HPV infection improved significantly, there was no significant improvement in attitudes toward HPV vaccination. This finding is consistent with our results, where attitudes toward HPV vaccination did not show a significant change post-intervention in the gender-specific group, even though knowledge increased. Flood et al. (59) also emphasized the potential of school-based interventions in improving HPV knowledge and vaccination intentions among middle adolescents (15–17 years). Their review highlighted that although educational interventions significantly improved knowledge and intentions, only a few studies actually measured changes in HPV vaccination uptake. This suggests that while knowledge and intentions are critical steps, they may not directly translate to higher vaccination rates without additional behavioral or systemic interventions. Our study similarly found improvements in knowledge and intentions, but also showed an actual increase in vaccination uptake, especially in GN interventions, reinforcing the importance of comprehensive strategies.

Despite GN strategies demonstrating a higher point estimate (5.7% vs. 2.5%) in increasing actual HPV vaccination uptake, the subgroup analysis did not yield a statistically significant difference between GN and GS strategies (*p* = 0.153). This non-significant finding suggests that although GN approaches may have greater potential to reach a broader audience (58, 59) and foster inclusivity, further high-powered studies are required to determine whether GN interventions consistently outperform GS interventions in boosting vaccination rates. Notably, GN interventions are comparable to provider-based interventions, which have been shown to improve uptake by 5–10% (60, 61). In contrast, GS interventions achieve only about half of this improvement. This highlights the importance of tailoring school-based interventions to be inclusive and gender-neutral to maximize their impact on vaccination uptake. In practice, educators and policymakers may weigh the broader coverage benefits of GN interventions against the potentially more tailored messaging in GS approaches. Ultimately, conclusive recommendations on implementing GN or GS strategies will depend on context-specific factors, such as available resources, cultural perceptions, and baseline vaccination rates.

It is important to note that some studies have reported more modest improvements or even null effects of GN strategies, particularly in settings where vaccine misinformation or cultural stigma surrounding HPV vaccination is prevalent (53, 62). These nuances highlight that while GN approaches may have a broad appeal, their success is heavily context-dependent and may require further adaptation to local cultural norms and acceptance of sexual health education.

The limitations of this study include potential publication bias, heterogeneity in study designs and interventions, and reliance on self-reported data for some outcomes. The high heterogeneity in knowledge outcomes suggests variability in educational methods and settings. Although we hypothesized that educational settings (secondary schools vs. colleges/universities) could explain some of the observed heterogeneity, a formal subgroup analysis was not feasible given the limited number of eligible school-based studies in both the GN and GS groups (fewer than four studies per subgroup). This limitation underscores the need for more research in diverse educational contexts to better elucidate setting-specific effects on HPV vaccination knowledge and outcomes. Furthermore, the lack of long-term follow-up in some studies limits the understanding of the sustained impact of these interventions on vaccination uptake and intentions. This underscores the need for more standardized and methodologically rigorous studies to ensure the reliability and applicability of the findings. Additionally, the limited number of RCTs and the lack of outcome separation by gender restrict our ability to analyze the specific impacts of GN interventions on male and female participants separately, which is crucial for tailoring public health strategies effectively.

The findings underscore the importance of implementing GN strategies in educational settings to improve HPV vaccination uptake. These strategies, by addressing a broader audience, can potentially lead to higher overall vaccination rates. Future research should focus on methodologically rigorous studies with long-term follow-up to better understand the sustained impact of these interventions. Additionally, exploring innovative educational methods, such as game-based learning, could further enhance the effectiveness of school-based health education programs. Understanding the context-specific factors that influence the success of these interventions, particularly in low- and middle-income countries, remains a critical area for future investigation. The implementation and success of HPV vaccination strategies, whether GN or GS, are influenced by broader contextual factors. Cultural attitudes toward vaccination and sexual health, socioeconomic disparities that limit healthcare access, and variable healthcare infrastructures can all mediate the impact of interventions. In LMICs, for instance, a lack of consistent cold-chain systems, inadequate health education frameworks, and sociocultural barriers may diminish the effectiveness of even the most robust school-based HPV programs. Future research should adapt interventions to these local contexts, ensuring that gender-neutral approaches are culturally sensitive and feasible within different economic and healthcare settings.

## 5 Conclusion

Our findings suggest that GN strategies, while demonstrating a potentially broader impact on HPV vaccination knowledge and intention, did not significantly outperform GS strategies in terms of actual vaccination uptake. Future studies should replicate these findings in larger, more diverse populations and with longer-term follow-up to definitively determine the comparative effectiveness of GN versus GS strategies.

## Data Availability

The original contributions presented in the study are included in the article/Supplementary material, further inquiries can be directed to the corresponding author.

## Footnotes

^1^https://www.rayyan.ai/, accessed on 7 June 2024
