# Supplementary material for: Gender-neutral vs. gender-specific strategies in school-based HPV vaccination programs: a systematic review and meta-analysis
Source: Front Public Health. 2025 Feb 18;13:1460511. doi: 10.3389/fpubh.2025.1460511 (PMC11876415; doi:10.3389/fpubh.2025.1460511)
Supplement: Supplementary file 1 [file Data_Sheet_1.docx]

**Search Strategies**

**Title: Gender-Neutral vs. Gender-Specific Strategies in School-Based HPV Vaccination Programs: A Systematic Review and Me-ta-Analysis

1. PubMed**

**Search Date:** 3 June 2024
**Search Limits/Filters:** Publication date from January 2006 to December 2023; English; Human studies
**Search Strategy (using MeSH and keywords where applicable):**

mathematica

Copy code

("human papillomavirus"[MeSH Terms] OR "HPV"[All Fields] OR "human papillomavirus"[All Fields])

AND

("vaccines"[MeSH Terms] OR vaccin*[All Fields] OR immuni*[All Fields] OR "vaccine uptake"[All Fields])

AND

("gender"[All Fields] OR "sex"[All Fields])

AND

("education"[MeSH Terms] OR "education"[All Fields] OR "school-based"[All Fields] OR "college-based"[All Fields] OR "university-based"[All Fields])

**Additional Steps:**

1. Filters applied in PubMed Advanced Search:
   - Publication dates: 1 January 2006 to 31 December 2023
   - Languages: English
   - Species: Humans
2. Retrieved references were exported into a reference manager (EndNote/Rayyan).

**2. Scopus**

**Search Date:** 3 June 2024
**Search Limits/Filters:** Publication date from January 2006 to December 2023; English
**Search Strategy (Title/Abstract/Keywords):**

vbnet

Copy code

TITLE-ABS-KEY( ( "HPV" OR "human papillomavirus" )

AND

( vaccin* OR immuni* OR "vaccine uptake" )

AND

( gender OR sex )

AND

( education OR "school-based" OR "college-based" OR "university-based" ) )

AND PUBYEAR > 2005

AND ( LIMIT-TO( LANGUAGE, "English" ) )

**Additional Steps:**

1. Publication years were restricted to 2006–2023 using the Scopus filtering options.
2. Results were exported to a reference manager to remove duplicates.

**3. Web of Science (Core Collection)**

**Search Date:** 3 June 2024
**Search Limits/Filters:** Publication date from January 2006 to December 2023; English
**Search Strategy (Topic Search in Web of Science):**

makefile

Copy code

TS=(

( "HPV" OR "human papillomavirus" )

AND

( vaccin* OR immuni* OR "vaccine uptake" )

AND

( gender OR sex )

AND

( education OR "school-based" OR "college-based" OR "university-based" )

)

AND

PY=(2006-2023)

AND

LA=(English)

**Additional Steps:**

1. Results were refined by Web of Science Categories and Document Types if needed (e.g., Articles, Reviews).
2. Records were exported into a reference manager for screening.

**4. Cochrane Library (Cochrane Central Register of Controlled Trials)**

**Search Date:** 3 June 2024
**Search Limits/Filters:** Publication date from January 2006 to December 2023; English
**Search Strategy:**

1. **Cochrane Library > Advanced Search > Search Manager**
2. Entered the following terms in the Search Manager lines:

less

Copy code

#1 (HPV OR "human papillomavirus"):ti,ab,kw

#2 (vaccin* OR immuni* OR "vaccine uptake"):ti,ab,kw

#3 (gender OR sex):ti,ab,kw

#4 (education OR "school-based" OR "college-based" OR "university-based"):ti,ab,kw

#5 #1 AND #2 AND #3 AND #4

1. **Date/Language Filters**: Publication from 2006 to 2023, English only (where possible).
2. Records were exported and duplicates removed.

**5. Other Sources**

1. **Reference Lists**: We screened the reference lists of relevant articles (primary studies, reviews, and meta-analyses) for additional eligible studies.
2. **Citation Tracking**: We used Google Scholar’s “Cited by” feature on key articles to identify any newer or related studies.
3. **Organization Websites**: We consulted websites of leading health organizations (e.g., WHO, CDC) for any relevant gray literature or reports.

**Notes on Filtering and Study Selection**

- **Date Restriction Justification**: Although our initial search filter was set from January 2000 to December 2023, we focused on 2006–2023 because the first HPV vaccine was approved in 2006. Studies published before 2006 were evaluated only if they included theoretical or preparatory interventions relevant to HPV vaccination.
- **Language Restriction**: We restricted to English to manage the scope of the review.
- **Screening Process**: Titles and abstracts were screened using Rayyan (<https://www.rayyan.ai/>). Full texts of potentially relevant articles were then assessed against our eligibility criteria (PICOS framework).
